# Habitat compression and ecosystem shifts as potential links between marine heatwave and record whale entanglements

**DOI:** 10.1038/s41467-019-14215-w

**Published:** 2020-01-27

**Authors:** Jarrod A. Santora, Nathan J. Mantua, Isaac D. Schroeder, John C. Field, Elliott L. Hazen, Steven J. Bograd, William J. Sydeman, Brian K. Wells, John Calambokidis, Lauren Saez, Dan Lawson, Karin A. Forney

**Affiliations:** 1 Department of Applied Mathematics, University of California, 1156 High Street, Santa Cruz, California 95064 USA; 2 Fisheries Ecology Division, Southwest Fisheries Science Center, National Marine Fisheries Service, NOAA, 110 McAllister Way, Santa Cruz, California 95060 USA; 3 Environmental Research Division, Southwest Fisheries Science Center, National Marine Fisheries Service, NOAA, 99 Pacific Street, Monterey, California 93940 USA; 4 Farallon Institute, 101 H Street, Suite Q, Petaluma, California 94952 USA; 5 Cascadia Research Collective, 218½ W 4th Avenue, Olympia, Washington 98501 USA; 6 Protected Resources Division, Southwest Regional Office, National Marine Fisheries Service, NOAA, 501 West Ocean Boulevard, Long Beach, California 90802 USA; 7 Southwest Fisheries Science Center, National Marine Fisheries Service, NOAA, Moss Landing, California USA; 8 Moss Landing Marine Laboratories, Moss Landing, California USA

**Keywords:** Ecology, Climate-change ecology, Ecosystem ecology

## Abstract

Climate change and increased variability and intensity of climate events, in combination with recovering protected species populations and highly capitalized fisheries, are posing new challenges for fisheries management. We examine socio-ecological features of the unprecedented 2014–2016 northeast Pacific marine heatwave to understand the potential causes for record numbers of whale entanglements in the central California Current crab fishery. We observed habitat compression of coastal upwelling, changes in availability of forage species (krill and anchovy), and shoreward distribution shift of foraging whales. We propose that these ecosystem changes, combined with recovering whale populations, contributed to the exacerbation of entanglements throughout the marine heatwave. In 2016, domoic acid contamination prompted an unprecedented delay in the opening of California’s Dungeness crab fishery that inadvertently intensified the spatial overlap between whales and crab fishery gear. We present a retroactive assessment of entanglements to demonstrate that cooperation of fishers, resource managers, and scientists could mitigate future entanglement risk by developing climate-ready fisheries approaches, while supporting thriving fishing communities.

## Abstract (web summary)

Climate-driven extreme events may have strong local impacts on marine organisms and fisheries. Here the authors report increased whale entanglements in the northeast Pacific following a marine heatwave, and propose compression of coastal upwelling habitat as the potential driver.

## Introduction

Due to unanticipated outcomes of climate change impacts on marine ecosystems, new challenges in management and conservation are arising^1^. One consequence of increasing anthropogenic climate warming is an increasing frequency, duration, and spatial extent of Marine heatwaves^2^ (MHWs). The variety of MHW impacts on marine life and fisheries has generated new challenges in ecosystem management and conservation of protected species^3–7^. Specifically, MHWs may lead to social and economic pressures, such as shifts in fisheries resources and/or by-catch of protected species^3,8–11^. In particular, whale entanglements in fishing gear have been increasing globally^12–14^, often at a rate greater than that of population recoveries from past exploitation, so there is a clear and immediate need to better understand how climate extremes are impacting habitat used by whales and fisheries^15–18^. Moreover, there is a growing need to improve the use and utility of ecosystem scientific advice relevant to marine resource management when confronted by novel ecosystem and fishery system states such as those that emerged in recent MHW events^19–22^.

The California Current Large Marine Ecosystem (CCLME) is a productive coastal upwelling ecosystem, where wind-driven upwelling brings enriched cool water to the surface that supports a diverse array of species and sustains important fisheries^23–25^. During 2014–2016, a MHW occurred in the North Pacific that resulted in an unprecedented multi-year warming event^5^. The impacts of the MHW were wide ranging^9,10^, but notably caused a sustained bloom of toxic *Pseudo-nitzschia* diatoms that led to the persistence of domoic acid (a neurotoxin impacting marine wildlife; e.g., shellfish poisoning^10,26–28^, record changes in biodiversity of pelagic species^29^, and an unprecedented delay in the opening of the commercial Dungeness crab (*Metacarcinus magister*) fishery in California (a fixed-gear trap fishery with vertical lines^6^). The crab fishery, which in recent decades has been among the largest by both volume and value in California^30–32^, normally opens in November and continues through mid-July, with catches peaking shortly after the initial opening and tapering to low levels throughout spring and early summer. However, high toxin concentrations during the 2015–16 fishing season led to the fishery opening being delayed until late March 2016. The MHW resulted not only in significant economic loss to fishing communities as a result of closures of shellfish and some finfish fisheries, but also coincided with an alarming rise in whale entanglements, mainly humpback whales (*Megaptera novaeangliae*), with crab fishing gear off California, sparking concern from fishers, resource managers, and conservationists^9,17,33^.

This study applies an ecosystem perspective to investigate links between the oceanographic conditions during the MHW and changes in the biodiversity and distribution of forage species, and how those changes promoted increased concentrations of whales within the primary area of the crab fishery. Seasonal and interannual variability of upwelling dynamics, nutrient supply, and forage species distribution within the CCLME are well understood^25,34,35^. In central California, during Spring–Summer, mesoscale upwelling habitat may be classified as the extent of cool water habitat (≤12 °C), as well as the development of upwelling fronts that enhance the mesoscale structure, supporting the development of primary and secondary consumer populations^36–38^. During years of strong upwelling conditions and sub-Arctic source water intrusions, the California Current is energized and cooler surface habitat extends further offshore, whereas during weaker upwelling years and increased sub-tropical source water, there is a reduction of enriched cool water habitat and upwelling fronts, and warmer offshore and/or sub-tropical water may intrude inshore^23,25,39^. Further, permanent geological features, such as submarine canyons, may act as thermal refugia, areas considered a suitable habitat for mid-water species and whales during ocean warming events^40,41^.

We hypothesize that onshore compression of the coastal upwelling ecosystem was at the root of the unusually high concentrations of whales occurring within the primary area of the crab fishery. Our hypothesis is summarized as the following sequence: (a) the MHW contributed to upwelling habitat compression, coinciding with the prevalence of domoic acid^10,28^, increases in epipelagic biodiversity due to the combined high abundance of warm- and cool-water species, and altered forage species availability (krill and anchovy abundance)^29,42^; (b) humpback whales exhibited prey-switching behavior and distribution shift in response to upwelling habitat compression-related changes in forage availability^43,44^, resulting in (c) an amplified spatial overlap of whales and crab fishing gear during 2014–2016. This overlap was intensified in spring 2016 when the opening of the crab fishery was delayed in response to domoic acid contamination in crabs such that it coincided with the migratory peak arrival of whales in the CCLME. An additional compounding factor is the long-term increase in recovering North Pacific humpback whale populations^45–47^; however, it is important to note there are multiple distinct population segments, as defined under the Endangered Species Act, of humpback whales within the CCLME, two of which are threatened or endangered and are of conservation concern^48^.

To assess the plausibility of our hypothesis, we synthesize information collected from an ecosystem assessment survey that monitors mid-water forage species distribution and biodiversity, whale occurrence, output from a data-assimilative oceanographic model used to develop an upwelling Habitat Compression Index (HCI), confirmed whale-entanglement records, and fishery landings data from the California Dungeness crab fishery. The evidence we present is consistent with our hypothesis that the MHW-induced upwelling habitat compression intensified the spatial overlap between whales and crab fishing in 2014–2016. We also summarize interactions between key stakeholders involved in the Dungeness crab fishery and whale-entanglement spike, including efforts by ecosystem scientists to provide expert advice during this record-intensity 3-year MHW. We propose a new framework for stakeholders to mitigate risk to protected species and fisheries. This framework calls for the development of a retroactive risk-assessment model involving easily observable stressors on the marine ecosystem. We discuss how monitoring ocean and forage species conditions, in conjunction with dynamic ocean management tools, may be used to develop seasonal risk assessments to mitigate whale entanglements, while maintaining an ongoing Dungeness crab fishery.

## Results and discussion

### MHW and habitat compression

**Fig. 1:**
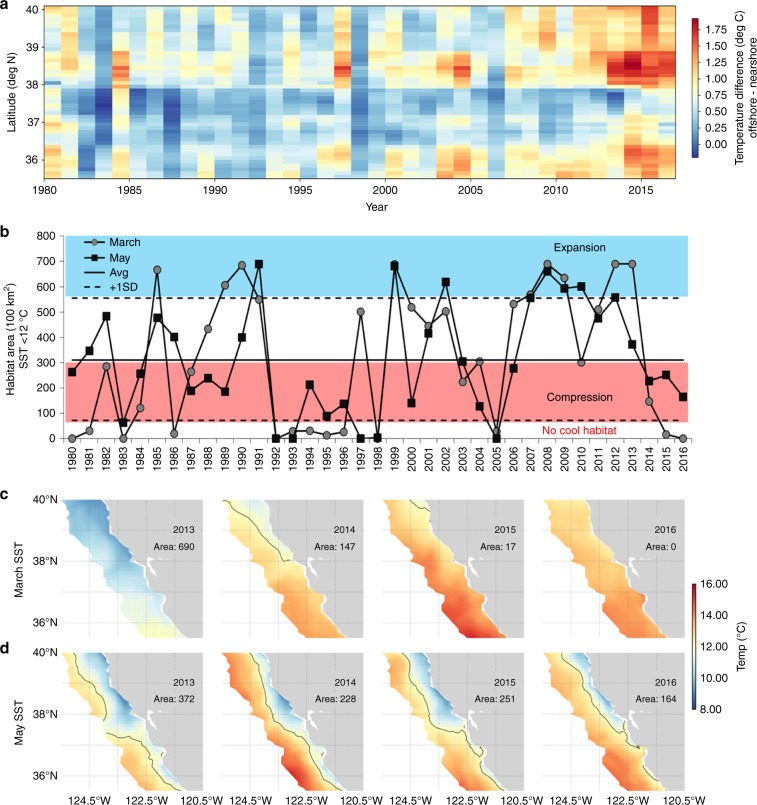
Upwelling habitat compression index, 1980–2016 (2 m surface temperature, derived from data-assimilative oceanographic model). **a** difference in onshore (0–50 km) and offshore (51–150 km) temperature gradients (average of March–May) as a function of latitude off California (**b**) time series describing change in area of cool 12 °C water during early March and May; the long-term mean and standard deviation provides basis for assessing the relative amount of cool surface water habitat and likelihood of cool water expansion vs. habitat compression, where values above + 1 SD indicate enhanced cool ocean conditions (i.e., La Niña or strong upwelling) and below −1 SD indicate no available cool habitat (e.g., El Niño or delayed upwelling); **c**, **d** spatial depiction of the change of cool water habitat during March and May during preceding (2013) and during (2014–2016) the marine heatwave and El Niño; the thin black line represents the 12 °C contour and area represents the number of pixels with values ≤12 °C. Source data are provided within the Source Data file.

Through application of the data-assimilative oceanographic model, we define a HCI to monitor changes in the areal extent of cool Sea Surface Temperature (SST; area of ≤12 °C), which allows for a long-term perspective on periods of either enhanced or decreased upwelling habitat (Fig. 1). Measuring the difference of SST between onshore and offshore provides a relative, but different, measure of cool water compression along the coast (Fig. 1a and Supplementary Figs. 1–3). During 1980–2016, in the months of March and May, several years were characterized by very little or no upwelling habitat; such as tropical El Niño years 1983, 1992, 1993, and 1997–98, and the delayed upwelling year of 2005, and most recently during the MHW period of 2014–2016 (Fig. 1b). Monitoring changes in the HCI during March to May is important due to the seasonal progression of prevailing upwelling winds from late winter to early spring as a primary driver of preconditioning of the marine ecosystem (Supplementary Figs. 1–3). The HCI in either month showed no long-term trend (*p* > 0.05), but time series displayed significant (type-1) autocorrelation at a one-year lag. Over nearly four decades, seasonal progression of the HCI ranged from low compression to very strong compression during March and then either a switch to more or less compression during May (Fig. 1b). This variability reflects the natural state of the upwelling ecosystem. The HCI during the MHW was not unprecedented and similar compression occurred during 1994–96 (Fig. 1b). However, the compression caused by the MHW is clear in either the HCI or as a function of latitude when comparing the offshore–onshore SST gradients (Fig. 1a, b). The latitudinal difference of offshore–onshore SST gradients indicates unprecedented compression (or lack of) of cold water (upwelling habitat) all along the CA coast both north of 38° N and south of 36° N during the MHW period (Fig. 1a), highlighting that upwelling habitat was limited to the nearshore northern part of the study region in March 2014, essentially absent in March 2015 and 2016, and compressed to nearshore areas in May 2014–2016 (Fig. 1c, d). Preceding the MHW, during 2013 (a year of record strong coastal upwelling and cool conditions^49^), the HCI indicates expansion of the cool SST area to above average conditions during March and May. During the MHW, in 2014, cool habitat was compressed in both months, but in 2015 and 2016 it was clear there was no cool habitat during March, and that cool habitat increased slightly during May owing to moderate upwelling^5,38,50^, but that cool habitat was compressed along the coast (Fig. 1b–d).

### Unusual biodiversity and changes in forage availability

**Fig. 2:**
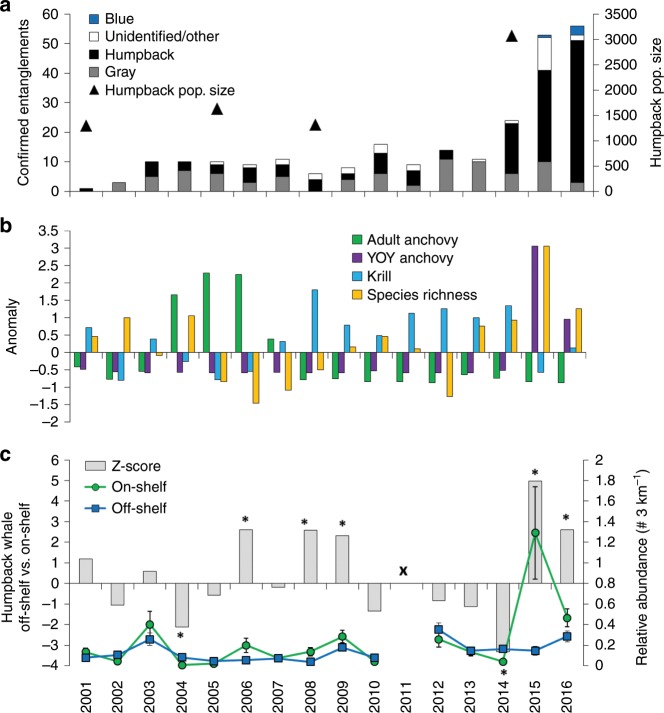
Changes in biological and ecosystem conditions in the California Current leading up to and during the marine heatwave period. **a** number of total confirmed whale entanglements per year detected off the US west coast, identified to species when possible, and estimates of humpback population size (data from NOAA^46^), **b** anomaly of abundance for total euphausiids (krill) and northern anchovy (adult and young-of-the-year; YOY), and total species richness within the central California region (standardized by mean and SD; 1990–2016; catch-per-unit-effort; CPUE; May–June); **c** summary of humpback whale relative abundance (per 3 km^−1^; mean and SD) during the annual ecosystem survey and assessment of changes in their occurrence On-Shelf (<200 m) vs. Off-Shelf (>200 m); bars represent *z*-score values and stars denote significant differences (*p* < 0.01); no whale survey data were collected in 2011 (denoted by X). See Supplementary Figs. 4–6 for additional information on changes in forage species abundance. Source data are provided within the Source Data file.

During the MHW, the CCLME experienced what can be now referred to as a “climate-stress test” on the ecosystem (Fig. 2). The impact of the MHW is observable in the anomaly of epipelagic species richness (Fig. 2b), which increased to record levels in 2015 and is attributed in part to a strong presence of sub-tropical and warm-water affinity species^29^. Increased epipelagic species richness in the CCLME during the recent MHW is now considered to reflect an anomalous ecosystem state, characterized by unusual abundance patterns and species assemblages, but with greater productivity than in previously documented warm years in which primary and secondary productivity were extremely reduced^51,52^. The anomaly of abundance time series (derived from mid-water trawls and standardized back to 1990 within long-term monitoring area; 36° N to 38° N) of the primary forage species used by humpback whales^53^ also indicates changes in forage species availability during the MHW. Total krill abundance, which exhibited strong positive anomalies between 2008 and 2014, was anomalously low in 2015, especially within the shelf region (Fig. 2b and Supplementary Figs. 4 and 5). Krill abundance within the long-term monitoring area was average during 2016 (Fig. 2b). Previous positive adult anchovy abundance anomalies occurred during 2004–2007 and were consistently negative throughout the 2008–2016 surveys, indicating that adult anchovy were not at previously high abundance levels in the CCLME (Fig. 2b). However, abundance anomalies for young-of-the-year (YOY) anchovy clearly indicate a major increase during 2015–16 and at the time were the highest recorded in the central California time series (Fig. 2b and Supplementary Figs. 4 and 6).

**Fig. 3:**
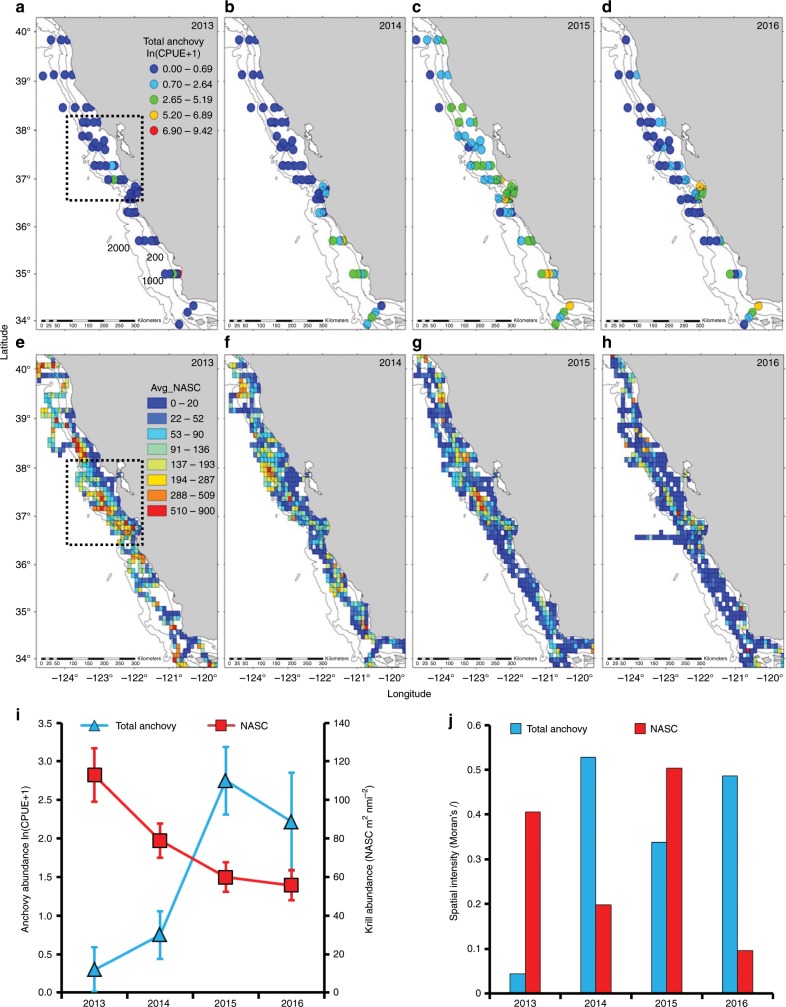
Changes in the distribution of forage species distribution off California in the year preceding (2013) and during the marine heatwave (2014–2016). **a**–**d** Mid-water trawl catches (catch-per-unit-effort; CPUE) of total northern anchovy; **e**–**h** acoustically determined (NASC; m^2^ nmi^−2^) krill distribution and abundance, averaged onto a 25 km^2^ grid. All survey data are derived from the Rockfish Recruitment and Ecosystem Assessment Survery (RREAS) during May–June. Dashed box indicates the long-term core sampling region of the RREAS; from Point Reyes 38° N to Monterey Bay 36.5° N. Map contours are the 200 m, 1000 m, and 2000 m isobaths (inshore to offshore; labeled in **a**). **i**, **j** Changes in the mean (error bars denote 95% confidence intervals) relative abundance of total anchovy (blue) and krill (red), and corresponding changes in spatial intensity and clustering (indexed by Moran’s *I*) for the mapped distributions per year. Source data are provided as a Source Data file.

Assessment of relative abundance and spatial intensity of forage species provides information for monitoring regional variability of prey resources used by whales at spatial scales relevant to whale movement and foraging patterns (Fig. 3 and Supplementary Fig. 4). Our evaluation of the regional distribution and spatial intensity of krill (measured by acoustics) and mid-water trawl catches of anchovy indicates changes in the availability of prey used by humpback whales preceding and during the MWH (Fig. 3). During 2013, when the HCI indicated expansion of cool water and record upwelling^5,38^ (Fig. 1), relative abundance of total anchovy was low, displaying low spatial intensity throughout the coast. In contrast, krill abundance was high, patches were plentiful, and spatial intensity was high, suggesting high clustering throughout the coast. During 2014, when the HCI indicated moderate compression of cool upwelled water, anchovy catches were significantly clustered in the southern portion of the coast, and krill abundance and spatial intensity declined (Figs. 1 and 3). At the peak of the MHW, under strong habitat compression (Fig. 1), 2015 catches of total anchovy were highly clustered coastwide, while krill spatial intensity decreased coastwide (Fig. 3). In 2015 and 2016, when upwelling habitat was highly compressed shoreward, krill abundance was lower, but spatial intensity increased in 2015 and declined abruptly in 2016, indicating there were fewer krill hotspots available for whales. Furthermore, mean abundance of total anchovy increased coastwide in 2015 and was relatively restricted in 2016 with extreme clustering within Monterey Bay and to the south off Point Conception (Fig. 3 and Supplementary Fig. 4). These indices provide reference points for evaluating potential thresholds in forage species availability utilized by whales.

### Rapid rise in whale entanglements

**Fig. 4:**
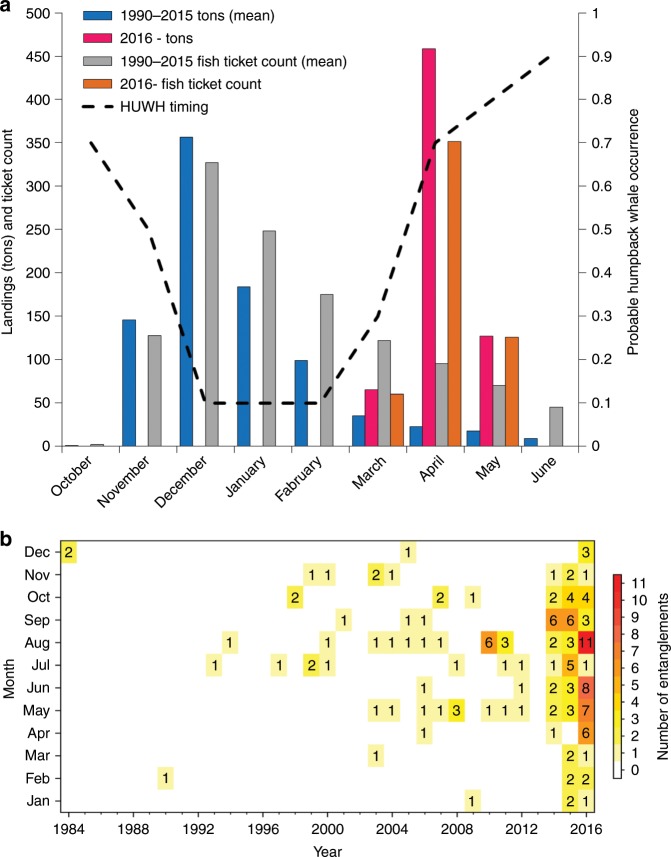
California Dungeness Crab fishery and humpback whale entanglements. **a** Summary of landings data by month for the California Dungeness Crab fishery; shown here as the long-term mean of 1990–2015 and the unusual delayed fishing season for 2016. Dashed line illustrates the assumed relative probability of occurrence for humpback whales (HUWH) off California reflecting their seasonal migration (derived from expert opinion, past surveys, and whale-watching activity). **b** Time series and summary of monthly confirmed humpback whale entanglements throughout the California Current illustrates the expansion of entanglements throughout the marine heatwave period (2014–2016). Source data are provided as a Source Data file.

The number of confirmed whale entanglements, most notably humpback whales, spiked throughout the MHW (Figs. 2a and 4). The confirmed whale-entanglement time series reflects a summary of reports for the entire US West Coast, but most entanglements were reported off California. A majority of the entanglements that were identified to some specific origin were linked to Dungeness crab fishing gear, especially within the central coast region of Monterey Bay where there is a substantial human population and a large whale-watching industry that could increase the relative probability of sighting entangled whales relative to less densely populated coastal areas^16,17^. When fishing gear is identified, the majority of confirmed humpback whale entanglements are due to pot gear and 70% is attributed to commercial Dungeness crab fishing gear^17^. Importantly, the reported entanglement location does not imply where the entanglement occurred, as whales have been documented to swim hundreds of miles trailing fishing gear for weeks, months, or even years^54^.

Confirmed entanglements of humpback whales clearly increased during 2014 and continued to increase during 2015 and 2016, while the MHW continued to influence the compression of cool water onshore (Figs. 1–2a, 4). Due to the summary of compiled entanglement reports and fishing activity, it was broadly perceived that increased entanglements were generally attributed to the Dungeness Crab fishery delayed opening during the 2015–2016 fishing season (Fig. 4a). However, the sharp increase in observed entanglements prior to the delayed opening indicated that this was only one of several factors, such that the increase in whale entanglements coincided with onset of the MHW in 2014, continued through 2015 (prior to the onset of the fishery delay) and then stayed at high levels throughout 2016. Confirmed humpback whale entanglements were reported in all months during the MHW period (Fig. 4b), indicating that unusually high numbers of entanglements were not isolated to a particular month or season. These changes suggest that ecosystem shifts and forage availability are a plausible, although unconfirmed, explanation for the increased entanglements in conjunction with the delayed fishing season. Humpback whales typically migrate to breeding grounds in the tropics during late fall and back to the CCLME in early spring^55^, meaning the peak timing and concentration of humpback whales and Dungeness crab fishery activity are typically offset (Fig. 4a). However, due to the delayed crab fishery opening in the 2015/2016 season, the peak in fishing activity was shifted and coincided with expected peak whale arrival and abundance off CA, placing whales in areas of fishing gear concentrations (which are typically set from the coast out to about the 130 m isobath^16^) in direct overlap with April–May fishing intensities (as indicated by fish ticket counts) that, to our knowledge, had never been observed before (Fig. 4 and Supplementary Fig. 7). In fact, our ecosystem assessment survey had never encountered such substantial concentrations of crab gear as it did in April–June of 2016, so effort was made to map gear locations to minimize impacts to the survey’s trawling activities (Supplementary Fig. 8).

### Whales follow their prey

Foraging, diet, and distribution studies indicate humpback whales are flexible foragers that perform rapid distribution changes in response to prey abundance and aggregation intensity, and switch from feeding on krill to schooling fish^43^. To illustrate a relative distribution shift of humpback whales, visual survey data collected during the ecosystem survey (May–June) were partitioned between on-shelf vs. off-shelf to examine where whales were more frequently encountered (Fig. 2c). Although humpback whales have shifted onshore and offshore in the past, the shift during the MHW was pronounced and statistically significant (*p* < 0.01; Fig. 2c), with a clear switch from concentrating offshore in 2014 to onshore during 2015–2016 (as indicated by *z*-scores). The measured changes in abundance and spatial intensity of krill and anchovy suggest humpback whales may have shifted from feeding offshore on krill to inshore to feed on anchovy^43^. Previous studies, using decades of survey data, revealed that krill hotspots are concentrated along the shelf-break, while high anchovy catches are more likely to concentrate on the shelf^41^ (Supplementary Fig. 9). Interestingly, when whale entanglements spiked in 2015 and 2016, there was a marked decline in krill and increase in anchovy abundance (Fig. 3), suggesting that forage availability for humpback whales was limited to the concentration of anchovy observed within Monterey Bay (Figs. 2–3 and Supplementary Fig. 4), where crab gear concentrations were substantial (Supplementary Fig. 8). Not seeing the onshore switch by whales in spring 2014 may indicate that availability of krill was still sufficient (Fig. 3), even though some whales did occur in shallow waters at that time (Forney Pers. obs). Further, entanglement records showed an uptick starting in the fall of 2014, as fishing gear was deployed on schedule, indicating that whales likely increasingly shifted onshore later in 2014.

Prey-switching by humpback whales is a complex behavior that is in part related to forage species abundance and patch distribution^43^. Specifically, stable isotope analysis of humpback whales over an extended time period supports prey-switching between krill and anchovies, in a pattern consistent with the shifts in abundance observed in the survey data^37,43^. Prey-switching behavior could also depend on the abundance of whales within a feeding ground, because whales may compete for the most profitable foraging areas and higher whale densities could result in some whales having to take up other foraging areas (e.g., nearshore). Whales require sufficient prey concentrations to meet their energetic demands and arrangement of prey aggregations is a critical aspect of their foraging and movement ecology. Therefore, spatial intensity of forage aggregations is relevant for understanding whale feeding behavior (e.g., movement, feeding attempts) and their relative abundance to resolve whether a feeding location is more or less profitable. When whales feed on krill patches, it is presumed that feeding is energetically conservative because krill patches are typically densely concentrated over several kilometers, concentrated at particular depths (e.g., pycnocline), and compared with anchovy, krill are less likely to evade capture by whales. When feeding on anchovy, whale foraging and feeding behavior is considerably more active and acrobatic (e.g., breaching behavior to shoal fish), because they pursue faster moving fish schools^56^. It is thought, but not firmly established, that entanglement risk can be attributed to increased feeding-related movements by whales within areas of dense anchovy and high concentration of crab gear (i.e., density and number of vertical lines). To complicate things, anchovy are known vectors for concentrating and transferring domoic acid toxin to their predators. Therefore, domoic acid poisoning may have influenced behavior and health of whales feeding on anchovy^26,28^.

### A retroactive evaluation of risk

In hindsight, despite the severe socio-economic impacts associated with the extended fishery closure, fishery managers should have more rigorously evaluated the tradeoffs between the economic needs of fishers and the likely increased risk to protected resources associated with the timing of the delayed opening of the 2015–16 crab fishing season. The delayed opening ultimately led to an unusually high concentration of fishing gear being deployed in areas where thousands of whales were arriving to feeding grounds containing very little food (Figs. 2–4) that was concentrated in areas targeted by the crab fishery. Although the suite of MHW impacts were being routinely reported by the media and in scientific meetings and symposia (http://www.marineheatwaves.org/), there were limited mechanisms for integrating and conveying the cumulative ecosystem impacts across the diverse range of monitoring programs and surveys that might have provided fisheries managers with a more comprehensive understanding of potential interactions and consequences of MHW impacts on the coastal ecosystems. Had such mechanisms been in place, and the risk of a delayed crab fishery opening to migrating whales better understood, a decision may have been made to keep the Dungeness crab fishery closed in high-risk areas for entanglements during the MHW. Such mechanisms would have been especially valuable during the 2015–16 season that was closed during the period of domoic acid contamination of Dungeness crab.

We believe that this retrospective evaluation provides valuable lessons for both the future management of CCLME fishery resources, as well as other fisheries systems that are likely to be impacted by unusual climate impacts and stressors. Specifically, closer evaluation of environmental conditions (Figs. 1 and 2), coupled with improvements in gear technology (such as breakaway lines, better tracking of gear to minimize lost and ghost fishing gear, and innovative gear that may not require buoy lines), should also serve to reduce and mitigate the risk of the fishery to protected resources^14^, as well as the risk of fisheries closures to result in future severe socio-economic impacts to fishing communities^57^. The lesson learned has broad implications for other marine ecosystems experiencing increased whale entanglements—when a future MHW persists for years, decision support tools can inform evaluations aimed at preventing entanglements by limiting fishing to times or areas with minimal risk, or using alternative fishing practices. Optimistically, the impact of the MHW and rise of entanglements helped to usher in the development of a working group composed of commercial fishers, state and federal resource managers, conservationists, and scientists that are collaborating to prevent future whale entanglements (http://www.opc.ca.gov/whale-entanglement-working-group/).

### Finding a solution requires collaboration

**Fig. 5:**
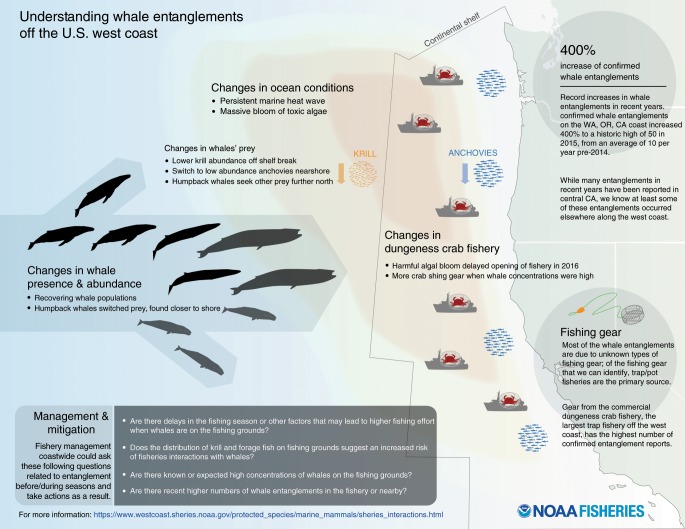
Summary of oceanographic and ecosystem changes within the California Current during the marine heatwave. The synthesis of information on changes in ocean and forage species described in this study provided the basis for establishing an ecosystem oceanographic perspective to support the California Dungeness Crab Whale Entanglement Working Group to form and further develop the Risk Assessment and Mitigation Program (RAMP) to reduce whale entanglements in fixed-gear fisheries off California. Infographic prepared by S. Kim.

Maintaining sustainable fisheries requires enabling sustainable interactions between fisheries resources, the fishing communities that depend on those resources, and the governance or management system^58^; in other words, developing robust social-ecological systems^20,59,60^. Implementing a social-ecological systems approach to the whale-entanglement problem would require that managers enable fishers, fishery managers, scientists, and other stakeholders to collectively develop rules and processes that evaluate and manage the risk to both the livelihoods of the resource users and the well-being of protected resources. To this end, the formation of a whale-entanglement working group represents a partnership to evaluate and mitigate entanglement risk, prevent future entanglements and to educate the public (Fig. 5). The synthesis of ecosystem science described here was instrumental in diffusing roadblocks and helping to develop California’s Risk Assessment and Mitigation Program (RAMP; Fig. 5). The RAMP involves a series of seasonal risk assessments (pre-, mid-, late- and post-season) based on ecosystem and fishery factors relevant to the Dungeness crab fishery. These factors involve the tracking of whale entanglements, whale concentrations, ocean and forage conditions, and fishing dynamics (gear concentrations, fishing activity and Dungeness crab market value, and domoic acid delays at the start of the fishing season; http://www.opc.ca.gov/risk-assessment-and-mitigation-program-ramp/). Although climate change is contributing to uncertainty surrounding the impacts of MHWs on ecosystem resilience, preparing climate-ready fishery solutions that involve the streamlining of disparate biophysical and socio-economic data and models is a priority for sustainable fisheries management^14,20,22^. The success of whale-entanglement working groups such as the RAMP will depend on their cooperation and collaborative interdisciplinary effort.

### Implications for dynamic ocean management

The connection among marine climate change, persistence of elevated SST and thresholds underlying MHW identification are now well established globally and regionally^2^. The factors underlying MHW events may occur under different ocean-climate conditions, but they have similar impacts on marine ecosystems and the services they provide (e.g., decline in fishery yield, unusual mortality events, and by-catch). Our study applied a straightforward measure of the amount of cool water upwelling habitat and upwelling habitat compression and evaluated it for understanding changes in forage species distribution and whale entanglements. This measure of habitat area may benefit dynamic ocean management^61,62^, especially during MHW monitoring^2^. Along with other metrics of upwelling, primary production, and harmful algal bloom occurrence, the HCI should be considered for ecosystem monitoring in the CCLME and may be easily extended to other eastern boundary upwelling ecosystems. Although the HCI provides a relative measure of thermal habitat, other satellite-based metrics are rapidly evolving to quantify seascape heterogeneity and future research should evaluate their ecological significance^21,63^. Dynamic ocean management tools for protected species offer platforms for providing custom-tailored information for managing and minimizing adverse impacts on sensitive species^62^, and should be explored for mitigating whale-entanglement risk. The benefit of these management tools is their flexibility, and extending the HCI with additional satellite-based seascape metrics that are spatially-explicit for krill, anchovy, whale distributions, fishing activity, and whale-entanglement risk can and should be investigated.

### New challenges for ecosystem-based management

Climate change and increased variability and intensity of climate events—in combination with recovering protected species populations and highly capitalized fisheries are posing new challenges for fisheries management, as demonstrated here by the rapid increases in whale entanglements. Long-term climate change is predicted to alter coastal upwelling ecosystems and changes are already recognizable^1^. Climate change is leading to more frequent crossing of temperature thresholds in the ocean that are likely to result in increased ecosystem variability and novel ecosystem consequences^2^, suggesting there may be no historical analogs for predicting future climate change impacts on marine ecosystems^64^. As rates of surface warming continue to rise globally, the ability of upwelling ecosystems to support healthy food-webs and fisheries is threatened. As warming oceanic water continues to impinge upon cool upwelled waters, compressing it closer to the coast and driving offshore species onshore, leading to shrinking habitat for whales and humans, we are likely to see increased socio-economic conflict with wildlife^65^. If increasing anthropogenic climate warming of the ocean is paired with increasing variability in coastal upwelling, an increase in compressed upwelling habitat may serve as a possible scenario for future climate change impacts on the CCLME and other eastern boundary upwelling systems. Thermodynamic warming of the global oceans dominates where dynamic processes are weak, and for the CCLME, dynamic processes are exceptionally strong in nearshore waters because of intense seasonal coastal upwelling in spring/summer^25,36^. For offshore and fall/winter, dynamic processes are weak throughout the CCLME^25^. As long as dynamic processes remain strong, we would predict that offshore SST would continue to rise in response to continued increases in thermodynamic warming related to increasing greenhouse gas concentrations and the thermodynamic feedbacks they trigger (primarily water vapor feedbacks).

Inter-decadal changes in fishing opportunities are already apparent in the CCLME. Many fishers, particularly small vessel fishermen, target a mix of Chinook salmon (*Oncorhynchus tshawytscha*), Dungeness Crab, albacore tuna (*Thunnus alalunga*), and groundfish (*Sebastes* spp.), and may shift their effort disproportionately from one fishery to another when one or more of those resources are less available^31,57,66^. For example, salmon fishing opportunities off California have progressively declined over the past few decades (including a fishery closure in 2008^67^), causing fishers to concentrate on other fishing opportunities, such as Dungeness crab fishing, that have been considerably more reliable^31,57^. These new management challenges can be overcome through synthesis of data-driven ecosystem and socio-economic assessments. The biophysical observation record and availability of ocean–ecosystem models in the CCLME is extensive, providing a wealth of information to develop robust management and sustainable fisheries. We relied on one ecosystem assessment survey to monitor biophysical changes, but greater insight will be gained through the integration of multiple surveys (using consistent methodology) to forecast climate-driven food-web alterations within the strongly seasonal and dynamic CCLME. Future analyses should explore evidence for spatial distribution shifts of coastal pelagic forage species and prey-switching behavior of predators to better understand and forecast ecosystem shifts. Furthermore, the collation, synthesis and maintenance of ecologically-relevant data streams is critical for guiding fishery management decisions and is now a major priority for mitigating whale-entanglement risk. The synthesis described in this study may be used to develop climate-ready fisheries approaches to minimize entanglement risk to whales, while supporting thriving fishing communities.

## Methods

### Habitat compression index

Monthly sea surface temperature conditions (at 2 m) and variability were obtained from historical reanalysis of ocean state derived from a data-assimilative oceanographic reanalysis model^68^. The oceanographic model is maintained by the University of California Santa Cruz (http://oceanmodeling.pmc.ucsc.edu/) and provides data from two different time spans 1980–2010 and 2010–present, with both analyses sharing the same grid (0.1° in the horizontal and 42 terrain following σ-levels in the vertical) but having different surface forcing^69,70^. The data-assimilative model has been extensively evaluated and integrates near real-time observation information from satellites (SST and altimetry) and from historical oceanographic survey data (e.g., Conductivity-Temperature-Depth casts). In this study, the use of a model is preferred over the use of satellite observation, because it assimilates observations and does not suffer from missing data from cloud contamination. Previous studies have indicated the role of late-winter seasonal upwelling (preconditioning^39,71^) and climate variability on the spring/summer abundance and distribution of krill and anchovy, micronekton biodiversity and top predators off California^29,40,44,72^, so here we focus on evaluating the HCI during March and May (although all months are preserved in the calculation of HCI). Standardized time series were examined for trends and autocorrelation to identify cycles of variability. Further, as part of a natural experiment, we assess the utility of the HCI to demonstrate how the MHW impacts the area of cold water and how it may have exacerbated the prevalence and persistence of domoic acid, altered forage species distribution patterns and increased the spatio-temporal overlap of whales and fishing gear.

Derivation of the HCI is straightforward. In eastern boundary upwelling ecosystems the spatial footprint of cool upwelled water is regularly demarcated by the differential boundary of warmer oceanic water offshore from cooler coastal water^24^, with upwelling conditions varying with latitude^73^. Therefore, the goal of the HCI is to track the area of cool surface waters as an index of potential upwelling habitat for assessing the spatio-temporal aspects of upwelling. Upwelling patterns of cold nutrient-rich water are clearly assessed by models and satellite observations and classified spatially by monitoring SST values ≤12 °C^36,39,50^. The HCI tracks the amount of area, determined by the number of grid cells in the model with 2 m surface temperature values of 12 °C or less; therefore, the time series reflects the area of cool water adjacent to the coastline and provides a measure for how compressed cool surface temperatures may be in a particular month. In this study, we extracted modeled 2 m temperature fields over the domain of 35.5–40 °N for each month and tracked the amount of area with temperature values ≤12 °C, resulting in monthly time series during 1980–2016. Cool expansion periods are defined as months with areas exceeding +1 SD of the full time series, limited cool habitat where area of cool water is less than −1 SD, and periods of habitat compression when the area of cool water falls between the long-term monthly mean and −1 SD. As upwelling conditions vary with latitude within the CCLME^73^, we also examine the variability of habitat compression as function of latitude by deriving the difference in surface temperatures between onshore and offshore per 0.1° of latitude. March–May monthly values of 2 m temperature extracted from the data-assimilative model are area averaged into onshore bins (0–50 km from shore) and offshore bins (50–150 km from shore). The onshore bin is subtracted from the offshore bin time series, with larger values denoting the offshore region having higher surface temperatures than the onshore region.

### Entanglement data

Whale-entanglement reports are compiled and maintained by NOAA Fisheries. The NOAA West Coast Region reviews all incoming documentation from entanglement reports (e.g., photo, video, descriptions, follow-up sighting reports, and response from disentanglement teams) before confirming them. Recent confirmed whale-entanglement data were derived from: https://www.westcoast.fisheries.noaa.gov/publications/protected_species/marine_mammals/5.2.2018_wcr_2018_entanglement_report_508.pdf). Considerable effort is required to assess each entanglement and determine the gear type that is involved. It is also important to note that a reported whale-entanglement location may not reflect the location of where the entanglement occurred. Confirmed entanglements for humpback whales are summarized by month to examine changes over time.

### Ecosystem surveys and assessment

This study uses ecosystem oceanographic data derived exclusively from the NOAA-NMFS Rockfish Recruitment and Ecosystem Assessment Survey (RREAS), stored on NOAA-ERDDAP, and reported by the California Current Integrated Ecosystem Assessment. Since 1983, the RREAS operates late April through mid-June to assess ocean conditions and the abundance and distribution of epipelagic micronekton throughout the entire coast of California (species enumeration was standardized in 1990). Mid-water trawls are collected at fixed sampling stations during night using a modified Cobb mid-water trawl with a 9.5 mm cod-end liner; 15 min tows were made at each station with a headrope depth of 30 m. After each haul, all taxa were enumerated and relative species abundance was measured as catch-per-unit-effort (CPUE) per station. For a synthesis of the spatial distribution and temporal variability of micronekton and their ecosystem considerations see^29,52^. Standardized anomaly time series of total euphausiids and northern anchovy abundance (adult and YOY), and total species richness are derived from^29,37,42^. A tool for visualizing and exploring these ecosystem data is available (http://dev.axiomdatascience.com/?portal_id=46#default-data/5).

During daylight hours, the RREAS transits among hydrographic stations collecting acoustic and visual observations of seabirds, marine mammals and other incidental observations (e.g., fishing gear). Multi-frequency echosounder (Simrad EK-60) data are collected during the RREAS to map and index the relative abundance (Nautical Area Scattering Coefficient (NASC m^2^ nmi^−2^) and spatial distribution of krill hotspots^41,74^. Acoustic data on krill hotspots are derived from and dynamics described in^41,74^. For mapping purposes and to assess spatial aggregation intensity patterns, acoustic data are averaged onto a 25 km^2^ grid. Similarly, trawl catches of total krill and total anchovy CPUE are mapped to assess interannual patterns (Supplementary Figs. 5 and 6). The relative mean abundance and 95% confidence intervals were computed for each year (2013–2016) and spatial aggregation intensity of krill and anchovy were measured using a two-dimensional Moran’s*/*test over the full sampling domain of the RREAS. We hypothesize that as the MHW developed and persisted, the abundance, distribution and spatial intensity pattern of krill and anchovy displayed marked regional abundance and spatial organization changes that corresponded with changes in upwelling habitat compression.

RREAS visual surveys of seabirds and marine mammals are described and derived from the annual CalCOFI State of the California Current Report^42^. The relative abundance time series and distribution of humpback whales during 2001–2016 (no visual surveys were collected during 2011 survey) are examined here for testing distribution changes of whales on and off the continental shelf (<200 m) per year. Given the aspects of the sightings data (e.g., removing poor sea state and fog conditions) and high motility of whales^46^, we use a Mann–Whitney *U*-test to determine whether relative abundance was greater on- or off-shelf in a given year, acknowledging that additional tracking and behavior measurements are needed to better understand whale movements. We hypothesize that humpback whales on feeding grounds will generally shift distribution in response to changes in forage species abundance and spatial organization (patch arrangement). The relative strong pattern of krill hotspots located offshore along the outer slope (especially coinciding with submarine canyons) vs. high spatial association of anchovy concentrations on the continental shelf, as well as changes in seabird aggregations, has repeatedly been documented and their dynamics described and modeled using monitoring data from the RREAS^37,41^.

The RREAS has rarely encountered dense concentration of crab fishing gear during May–June and the survey was impacted during 2016 when substantial crab gear was encountered, causing the unforeseen challenges while conducting the mid-water trawl (i.e., gear avoidance in coastal waters). At that time, a decision was made to collect visual survey data using strip-transect methodology to describe the relative concentration and distribution of crab gear during the 2016 survey. Even with these efforts to map the distribution of crab gear, at least 12 trawls were cancelled, as no clear path through the gear could be discerned (out of 137 completed trawls), whereas in the previous 34 years of the survey a trawl had never been cancelled due to high crab gear densities. An example of sightings data for crab gear is provided (see Supplementary Fig. 8). However, it should be noted that the fishery does not require mandatory logging by fishers for reporting where gear is set and that visual surveys of fishing gear conducted by aerial surveys, due to their ability to cover broad areas rapidly are preferred, but are not always available.

### Fishery landings data

California Dungeness crab landing (metric tons) and fish ticket data were based on queries from the Pacific Fisheries Information Network (PacFIN). The California Dungeness Crab fishery typically opens in November and typically lasts into June or July, with both catches and the number of landings peaking in late fall and early winter, and declining into the spring. To assess the amount of crab landed by the commercial Dungeness Crab fishery, the total metric tons and number of fish tickets per month for all landings reported in the State of California was summarized. Landings data were aggregated as a long-term average for 1990–2015 and then separately for the delayed fishing season of 2016 (Supplementary Fig. 7). Fish ticket data were treated similarly, although these represent a relative, rather than absolute, number of trips or landings across the fishing fleet, as in some instances fishermen may report more than one fish ticket for a given trip, depending on markets and sales arrangements.

### Reporting summary

Further information on research design is available in the Nature Research Reporting Summary linked to this article.

## Supplementary information

Supplementary Information

Reporting Summary

## Data Availability

The source data underlying Figs. 1a, b, 2a–c, 3, and 4, and Supplementary Figs. 3 and 8 are provided as a Source Data file. All data pertaining to oceanographic conditions, ecosystem surveys, and whale-entanglement time series are freely available from the California Current Integrated Ecosystem Assessment (CCIEA): https://www.integratedecosystemassessment.noaa.gov/regions/california-current-region/index.html Oceanographic model output is freely available from: http://oceanmodeling.ucsc.edu/ccsnrt/ All data from the RREAS is maintained on the NOAA-ERDDAP portal and are freely accessible: https://oceanview.pfeg.noaa.gov/erddap/tabledap/FED_Rockfish_Catch.graph. A tool for visualizing, exploring, and accessing RREAS data is available: http://dev.axiomdatascience.com/?portal_id=46#default-data/5 Data on fishery landings are available from the Pacific Fisheries Information Network (PacFIN), retrieval dated 22 May 2018. Pacific States Marine Fisheries Commission, Portland, Oregon (www.psmfc.org). It is noteworthy that confidentiality restrictions prevent access to raw data in some instances. Filtered data are available at: https://reports.psmfc.org/pacfin/f?p=501:1000.

## Source data

Source Data
