# Supplementary material for: Habitat compression and ecosystem shifts as potential links between marine heatwave and record whale entanglements
Source: Nat Commun. 2020 Jan 27;11:536. doi: 10.1038/s41467-019-14215-w (PMC6985238; doi:10.1038/s41467-019-14215-w)
Supplement: Supplementary file 1 — Supplementary Information [file 41467_2019_14215_MOESM1_ESM.pdf]

Habitat compression and ecosystem shifts as potential links between marine heatwave and record whale entanglements

Jarrod A. Santora[*1, 2], Nathan J. Mantua[2], Isaac D. Schroeder[3], John C. Field[2], Elliott L. Hazen[3], Steven J. Bograd[3], William J. Sydeman[4], Brian K. Wells[2], John Calambokidis[5], Lauren Saez[6], Dan Lawson[6], Karin A. Forney[7, 8]

[*]Corresponding author email: jsantora@ucsc.edu

[1]Department of Applied Mathematics, University of California, 1156 High Street, Santa Cruz, CA 95064, USA
[2]Fisheries Ecology Division, Southwest Fisheries Science Center, National Marine Fisheries Service, NOAA, 110 McAllister Way, Santa Cruz, CA, 95060, USA
[3]Environmental Research Division, Southwest Fisheries Science Center, National Marine Fisheries Service, NOAA, 99 Pacific Street, Monterey, CA 93940, USA
[4]Farallon Institute, 101 H Street, Suite Q, Petaluma, CA, 94952, USA
[5]Cascadia Research Collective, 218½ W 4th Avenue, Olympia, WA 98501, USA
[6]Protected Resources Division, Southwest Regional Office, National Marine Fisheries Service, NOAA, 501 West Ocean Boulevard, Long Beach, CA 90802, USA
[7]Southwest Fisheries Science Center, National Marine Fisheries Service, NOAA, Moss Landing, CA, USA
[8]Moss Landing Marine Laboratories, Moss Landing, CA USA

Supplementary Figure 1

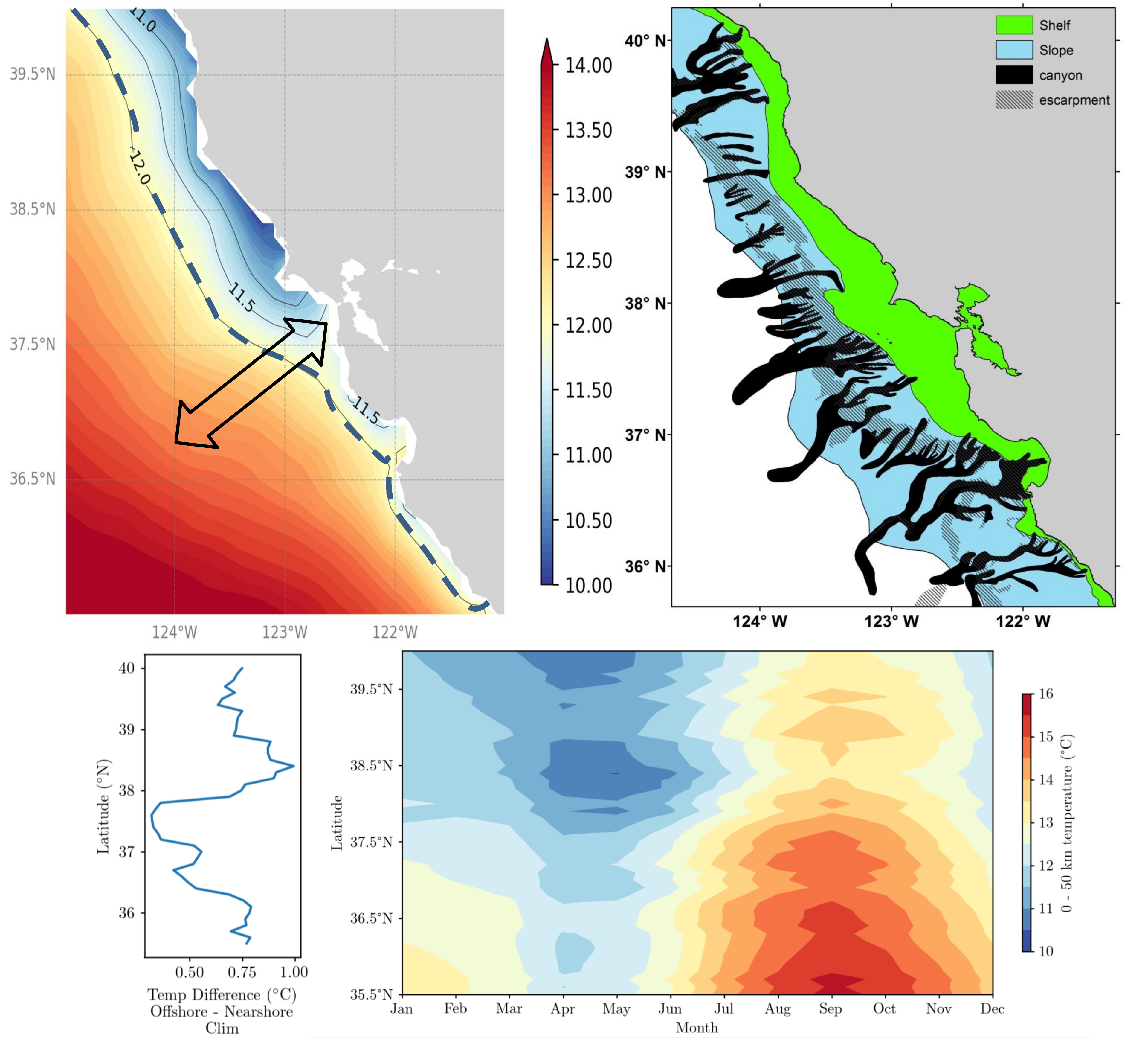

Supplementary Figure 1: (**left**) Climatological spatial mean of Sea Surface Temperature (SST) during March-May, 1980-2016, derived from the data-assimilative oceanographic model; hatched line indicates average location of the 12˚C isotherm and arrow indicates generalized movement of cool water expansion and compression along the coast. (**right**) detailed view of coastal geomorphology differences between the outer slope and shelf habitats, highlighting the concentration of submarine canyons, all of which influences distribution of epipelagic forage species, whales and fishing (**bottom**) onshore-offshore difference in SST (March-May) and monthly climatological mean nearshore SST by latitude over the study area. Source data available from: http://oceanmodeling.ucsc.edu/ccsnrt/

Supplementary Figure 2

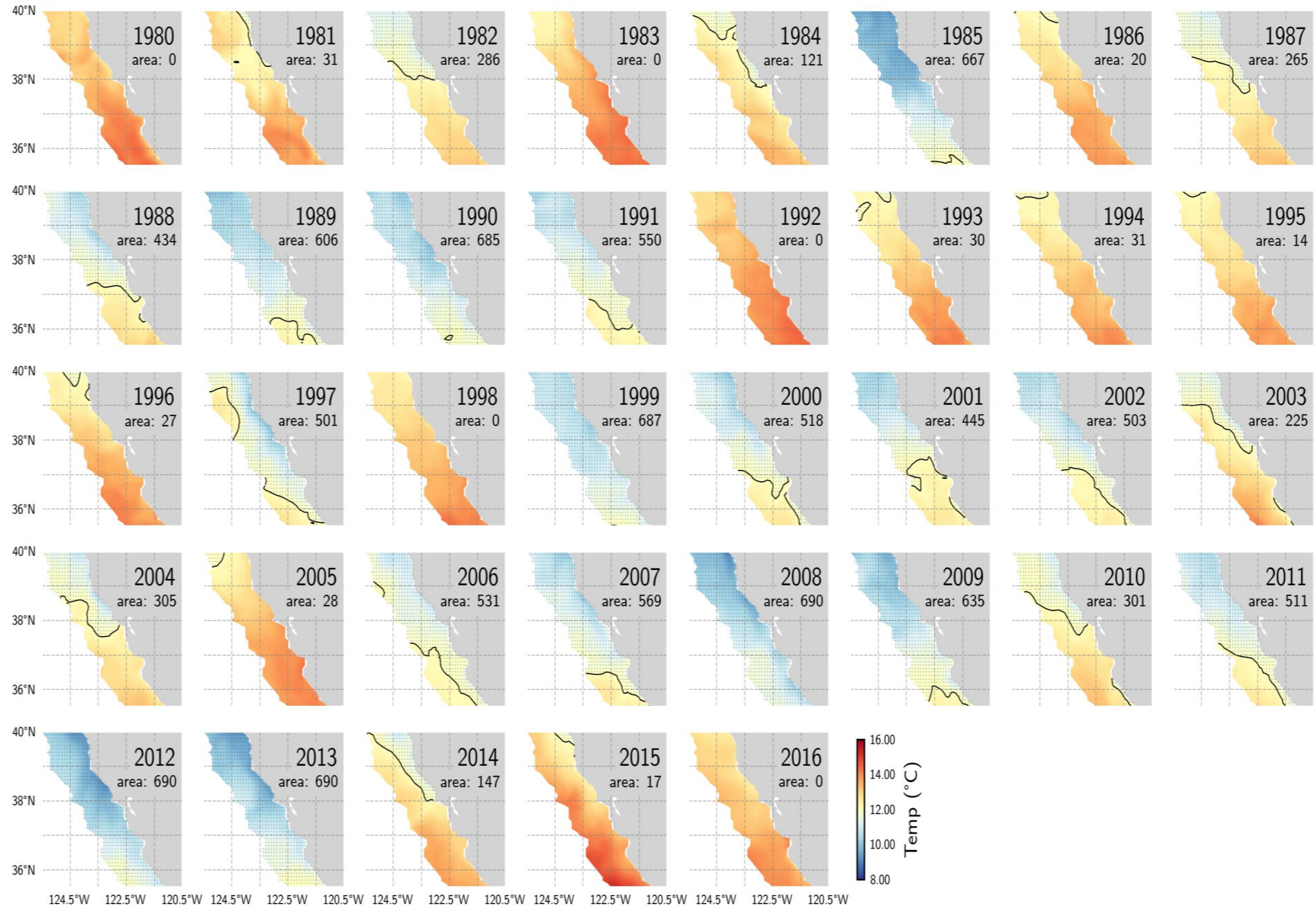

Supplementary Figure 2: Spatial representation of the Habitat Compression Index (HCI): March sea-surface temperature (SST), 1980-2016, derived from the data-assimilative oceanographic model illustrating periods of expansion and compression of cool water habitat; 'area' indicates the number of model grid cells where SST ≤12˚C. See Figure 1 for a time series of the HCI. Source data available from: http://oceanmodeling.ucsc.edu/ccsnrt/

Supplementary Figure 3

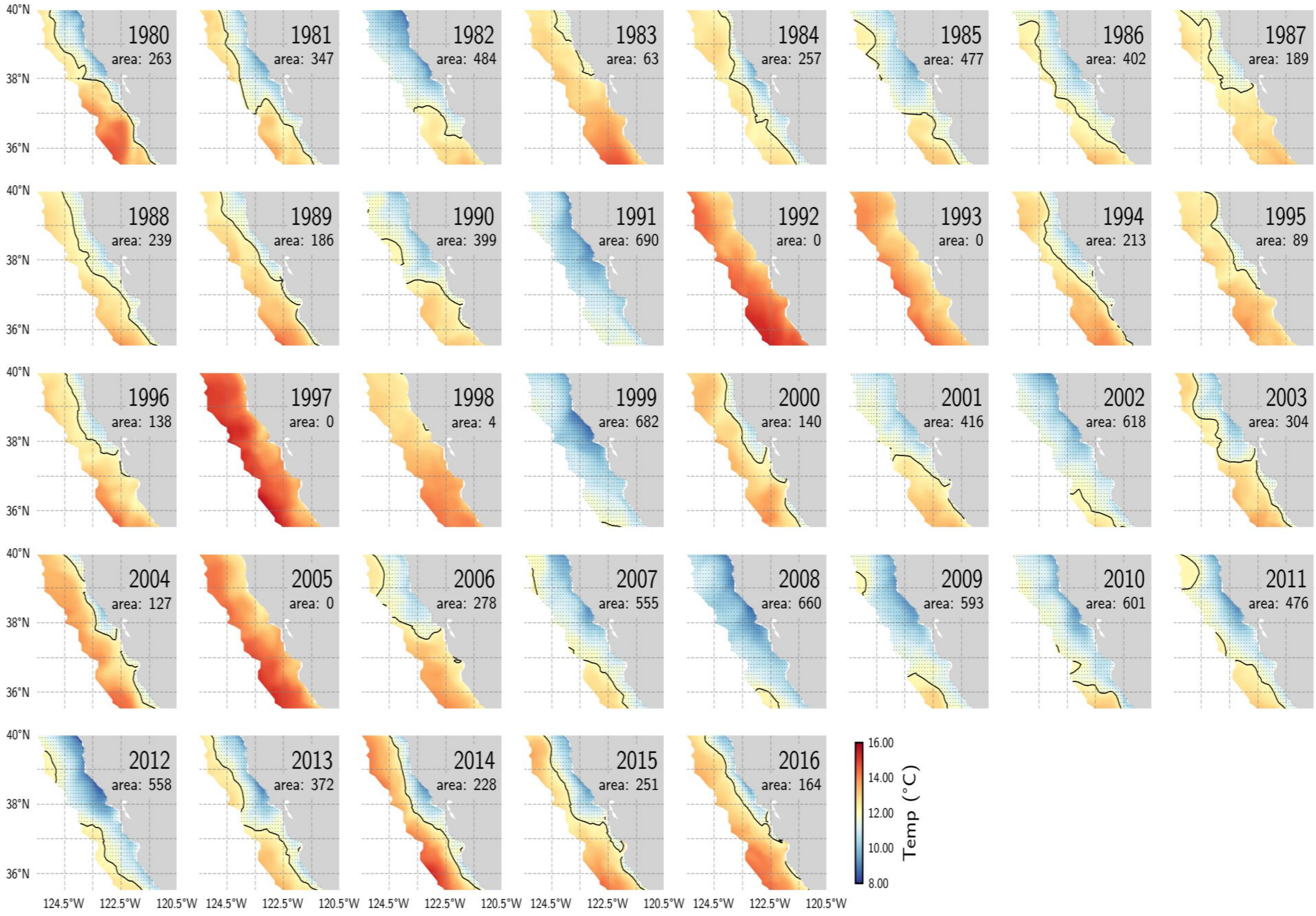

Supplementary Figure 3: Spatial representation of the Habitat Compression Index (HCI): May Sea-Surface Temperature (SST), 1980-2016, derived from the data-assimilative oceanographic model illustrating periods of expansion and compression of cool water habitat; ‘area’ indicates the number of model grid cells where SST ≤12˚C. See Figure 1 for a time series of the HCI. Source data available from: http://oceanmodeling.ucsc.edu/ccsnrt/

Supplementary Figure 4

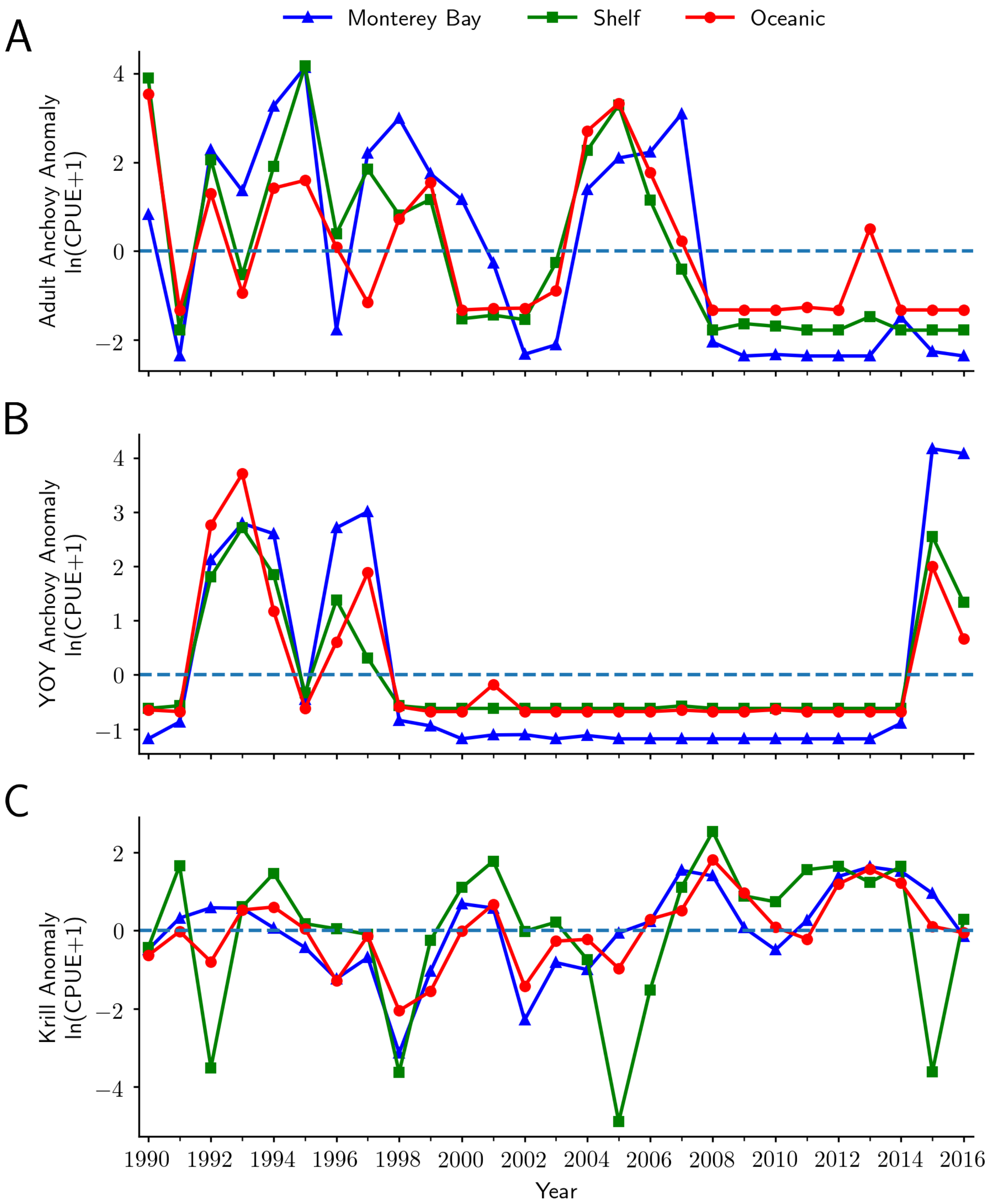

Supplementary Figure 4: Standardized anomaly of abundance (ln(CPUE+1)) per habitat area off central California (36˚N-38˚N; see inset in Figure 3) for: (A) adult anchovy, (B) Young-of-the-year (YOY) anchovy and (C) total krill. Dashed line indicates zero or neutral anomaly; 'Shelf' and 'Oceanic' series are sampling stations collected in waters less than and greater than 200 m, respectively, and 'Monterey Bay' includes all sampling stations within Monterey Bay. Habitat area and time series are defined in and updated from previous studies[1-2]. Source data available from Source Data File and https://oceanview.pfeg.noaa.gov/erddap/tabledap/FED_Rockfish_Catch.graph

Supplementary Figure 5

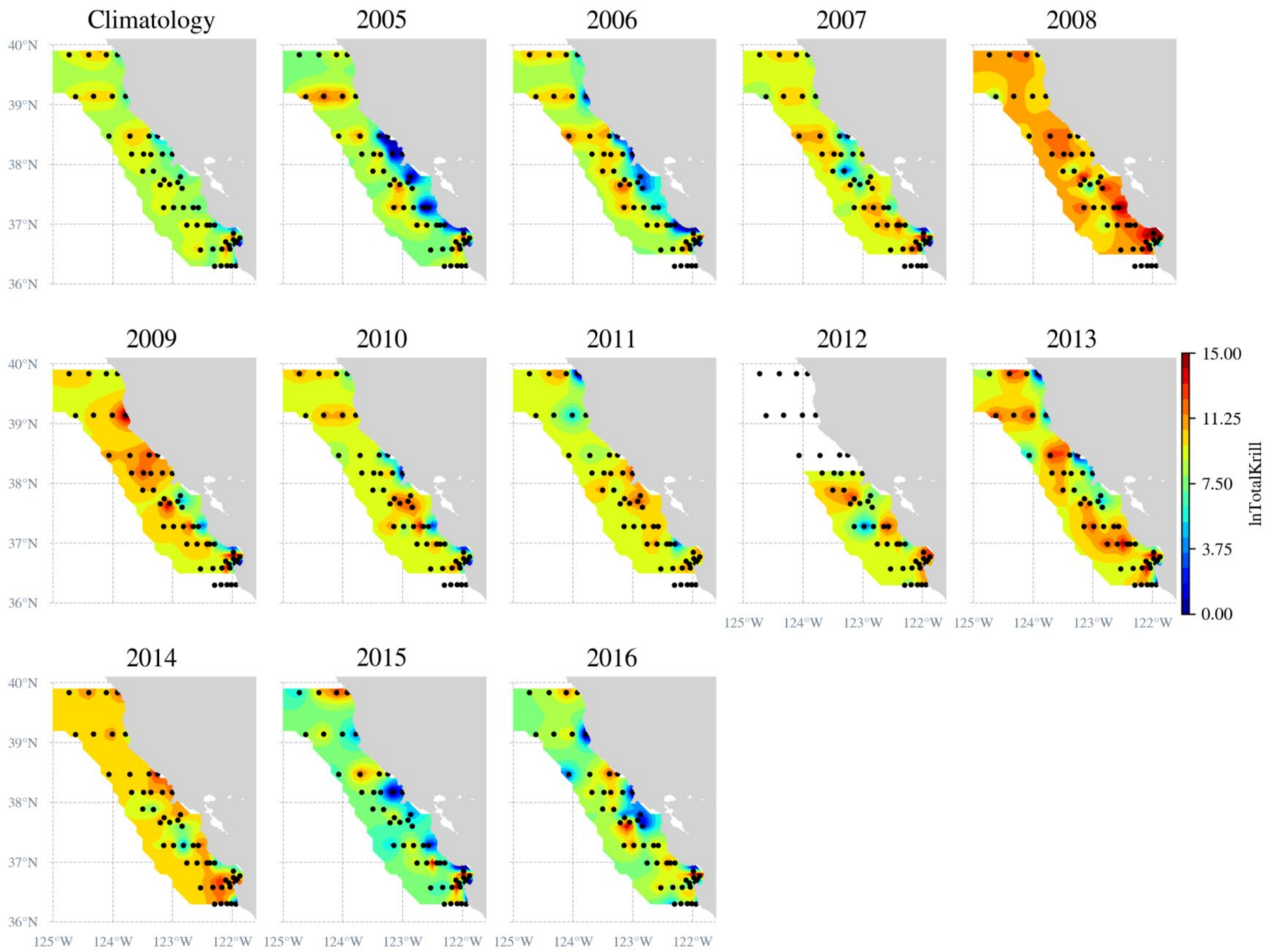

Supplementary Figure 5: Interpolated abundance of total euphausiids (ln(CPUE+1)) collected by mid-water trawl during the Rockfish Recruitment and Ecosystem Assessment Survey. Black dots are sampling stations. The climatology in the first panel illustrates the spatial mean of 1990-2016. Source data available from: https://oceanview.pfeg.noaa.gov/erddap/tabledap/FED_Rockfish_Catch.graph

Supplementary Figure 6

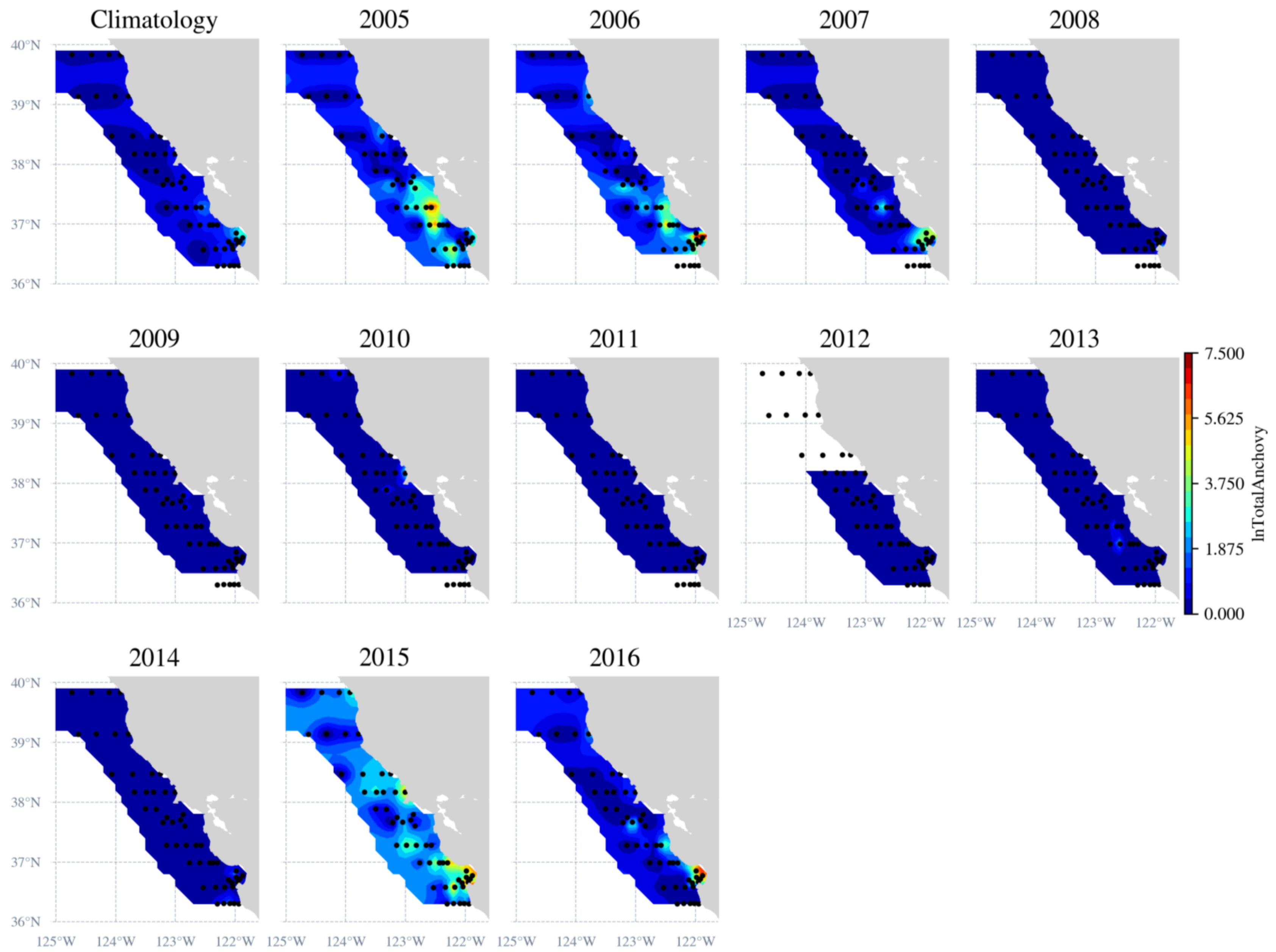

Supplementary Figure 6: Interpolated abundance of total (encompassing adult and young-of-the-year) northern anchovy (ln(CPUE+1)) collected by mid-water trawl during the Rockfish Recruitment and Ecosystem Assessment Survey. Black dots are sampling stations. The climatology in the first panel illustrates the spatial mean of 1990-2016. Source data available from: https://oceanview.pfeg.noaa.gov/erddap/tabledap/FED_Rockfish_Catch.graph

Supplementary Figure 7

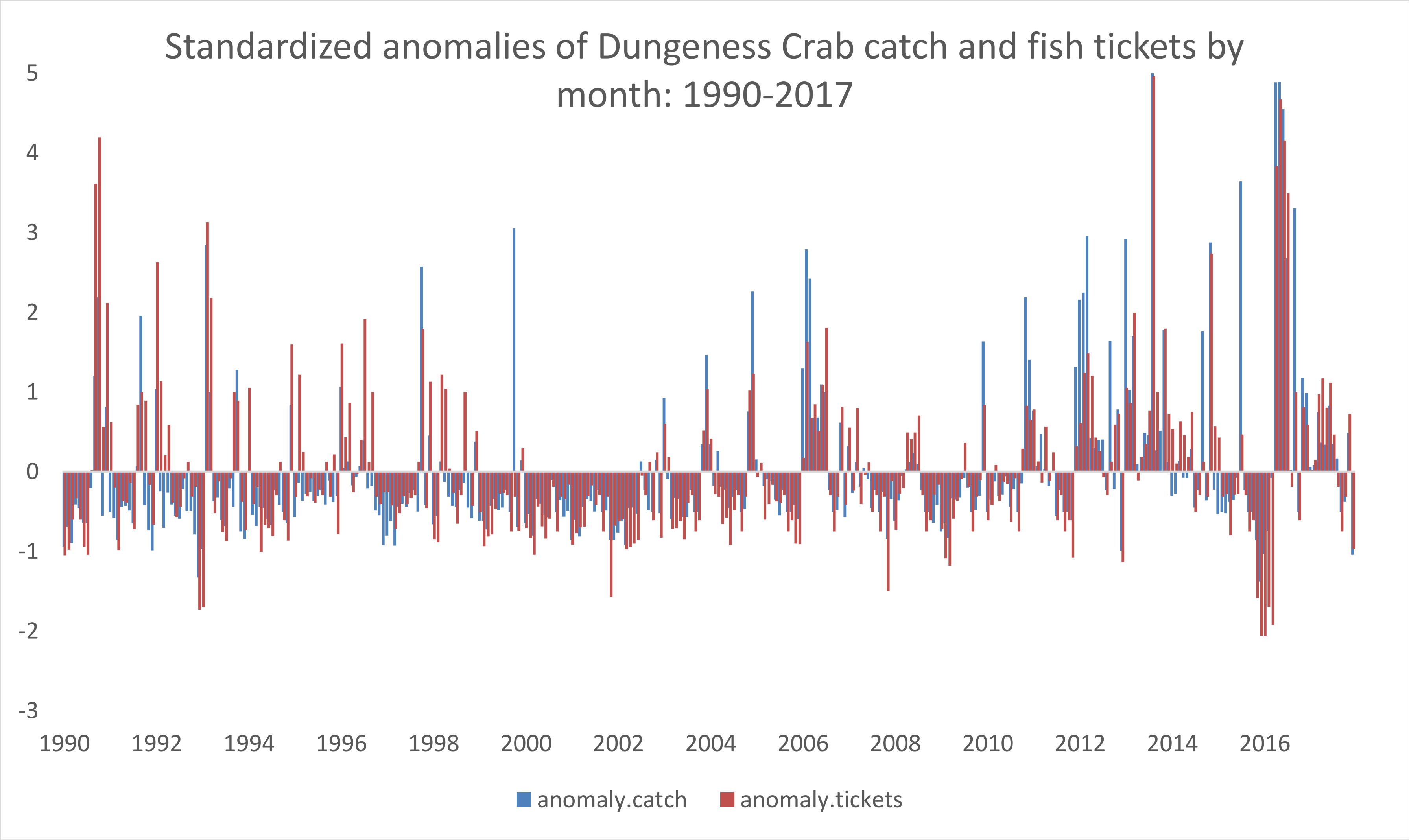

Supplementary Figure 7: Standardized anomalies of Dungeness crab catch and fish tickets off California by month, 1990-2017. Data on fishery landings is available from the Pacific Fisheries Information Network (PacFIN), retrieval dated May 22nd, 2018. Pacific States Marine Fisheries Commission, Portland, Oregon (www.psmfc.org). Note that confidentiality restrictions prevent access to raw data in some instances. Filtered data are available at: https://reports.psmfc.org/pacfin/f?p=501:1000:

Supplementary Figure 8

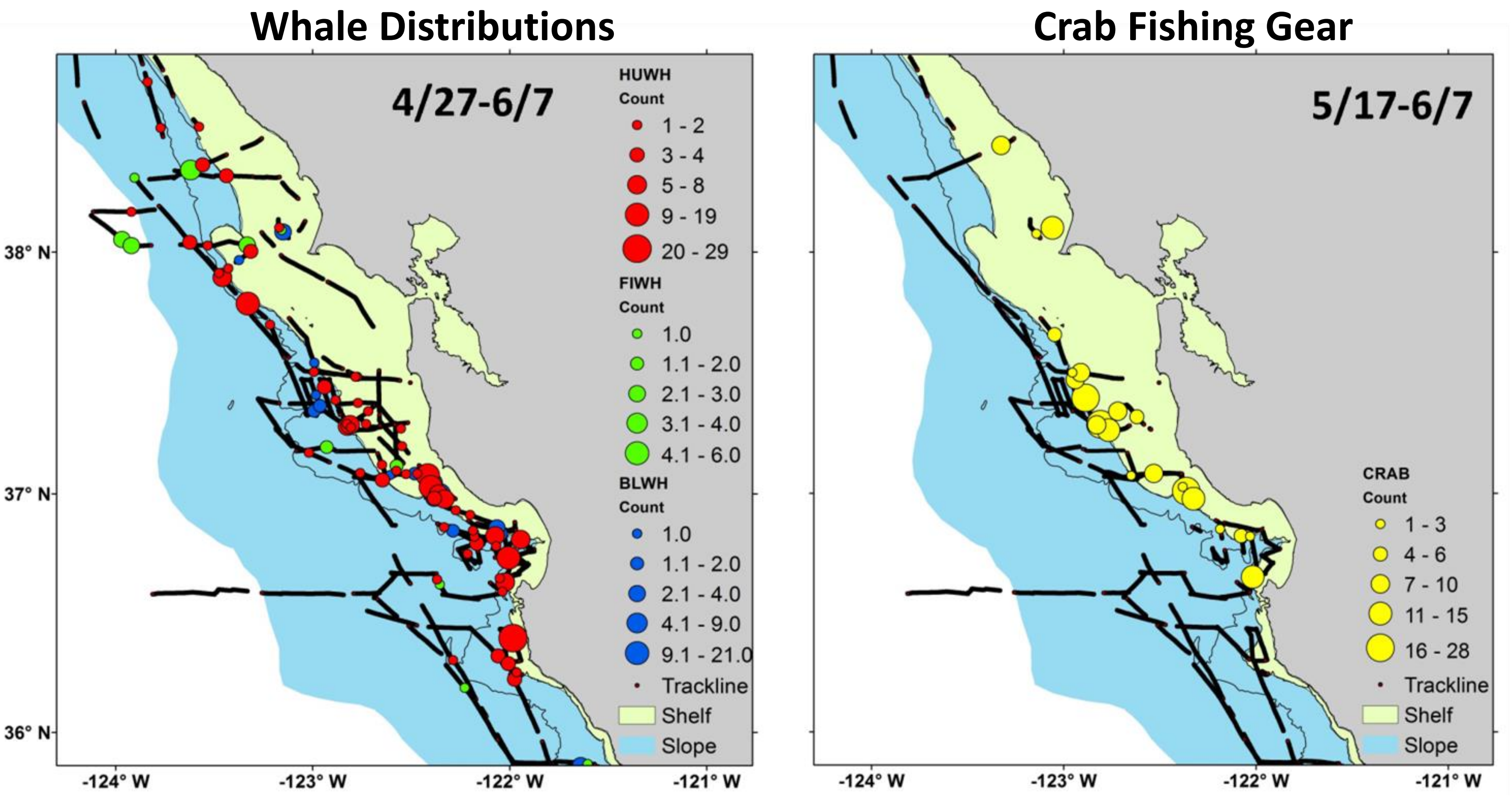

Supplementary Figure 8: Sighting distribution of (left) whales and (right) crab pot buoys collected during the 2016 NOAA Rockfish recruitment and Ecosystem Assessment Survey; HUWH (red) is humpback whale, FIWH (green) is fin whale, BLWH (blue) is blue whale; black line indicates survey trackline. Note due to the unusual circumstances of encountering crab gear, it was not systematically collected until mid-May 2016. Source data are provided as a Source Data file.

Supplementary Figure 9

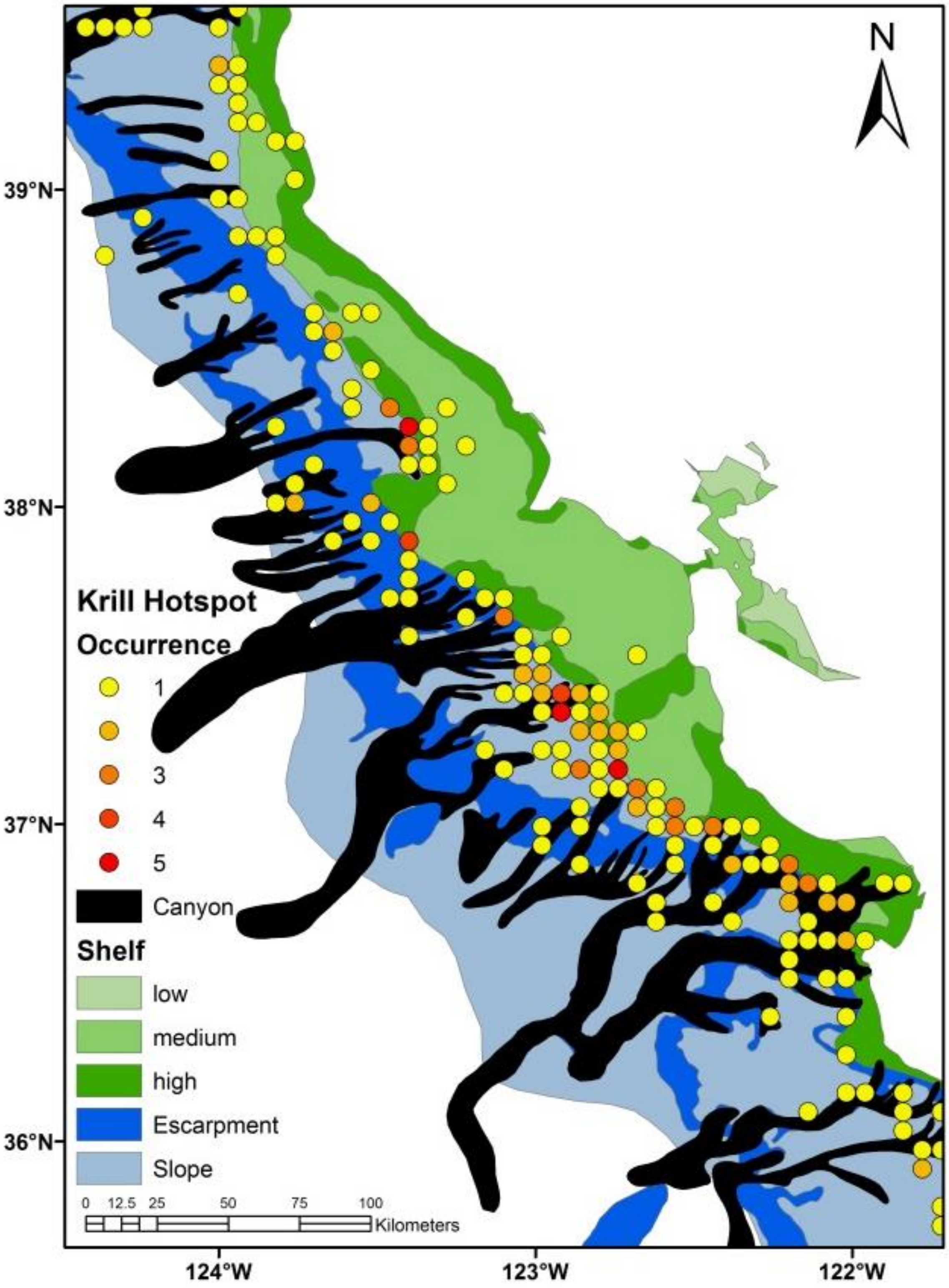

Supplementary Figure 9: Location and persistence of krill abundance hotspots mapped using acoustics during the Rockfish Recruitment and Ecosystem Assessment Survey[3]. The summary is based on surveys collected during 2001-2016. Krill hotspots are concentrated throughout the shelf-break region, especially near the heads of submarine canyons[3]. During low krill abundance years, fewer krill hotspots (i.e., whale foraging areas) are likely to occur and their spatial intensity is lower. Shelf classification (low, medium and high) reflects relief.

References:

[1] Santora, J. A., Schroeder, I. D., Field, J. C., Wells, B. K. & Sydeman, W. J. Spatio-temporal dynamics of ocean conditions and forage taxa reveals regional structuring of seabird-prey relationships. *Ecol. Appl*. **24**, 1730-1747 (2014).

[2] Santora J. A., Hazen, E. L., Schroeder, I, D., Bograd, S., J., Sakuma, K. M., & Field, J. C. Impacts of ocean climate variability on biodiversity of pelagic forage species in an upwelling ecosystem. *Mar. Ecol. Prog. Ser.* **580**, 205-220 (2017).

[3] Santora, J. A., Zeno, R., Dorman, J. G., & Sydeman, W. J. (2018) Submarine canyons represent an essential habitat network for krill hotspots in a Large Marine Ecosystem. *Scientific Reports* **8**(1). doi:10.1038/s41598-018-25742-9
