# Supplementary material for: Habitat compression and ecosystem shifts as potential links between marine heatwave and record whale entanglements
Source: Nat Commun. 2020 Jan 27;11:536. doi: 10.1038/s41467-019-14215-w (PMC6985238; doi:10.1038/s41467-019-14215-w)
Supplement: Supplementary file 2 — Reporting Summary [file 41467_2019_14215_MOESM2_ESM.pdf]

nature research

Corresponding author(s): DBPR: NCOMMS-19-19216

Last updated by author(s): Jun 28, 2019

Reporting Summary

Nature Research wishes to improve the reproducibility of the work that we publish. This form provides structure for consistency and transparency in reporting. For further information on Nature Research policies, seeAuthors & Referees and theEditorial Policy Checklist .

Statistics

For all statistical analyses, confirm that the following items are present in the figure legend, table legend, main text, or Methods section.

n/a	Confirmed	
x		The exact sample size (*n*) for each experimental group/condition, given as a discrete number and unit of measurement
	x	A statement on whether measurements were taken from distinct samples or whether the same sample was measured repeatedly
	x	The statistical test(s) used AND whether they are one- or two-sided<br>*Only common tests should be described solely by name; describe more complex techniques in the Methods section.*
	x	A description of all covariates tested
	x	A description of any assumptions or corrections, such as tests of normality and adjustment for multiple comparisons
	x	A full description of the statistical parameters including central tendency (e.g. means) or other basic estimates (e.g. regression coefficient) AND variation (e.g. standard deviation) or associated estimates of uncertainty (e.g. confidence intervals)
	x	For null hypothesis testing, the test statistic (e.g. *F*, *t*, *r*) with confidence intervals, effect sizes, degrees of freedom and *P* value noted<br>*Give P values as exact values whenever suitable.*
x		For Bayesian analysis, information on the choice of priors and Markov chain Monte Carlo settings
x		For hierarchical and complex designs, identification of the appropriate level for tests and full reporting of outcomes
x		Estimates of effect sizes (e.g. Cohen's *d*, Pearson's *r*), indicating how they were calculated

Our web collection on statistics for biologists contains articles on many of the points above.

Software and code

Policy information about availability of computer code

Data collection	No software was used for data collection. All data were originated from public archives and previous publications. Observations were collected aboard a fisheries research vessel and stored in a database (NOAA ERDDAP Server).
Data analysis	Data analysis was conducted using the statistical program RStudio (version 3.5.3) and maps were prepared with ESRI ArcView (version 10).

For manuscripts utilizing custom algorithms or software that are central to the research but not yet described in published literature, software must be made available to editors/reviewers. We strongly encourage code deposition in a community repository (e.g. GitHub). See the Nature Research guidelines for submitting code & software for further information.

Data

Policy information about availability of data

All manuscripts must include a data availability statement. This statement should provide the following information, where applicable:

- Accession codes, unique identifiers, or web links for publicly available datasets
- A list of figures that have associated raw data
- A description of any restrictions on data availability

The datasets generated during and/or analyzed during the current study are available from the CCIEA and NOAA-ERDDAP data repositories. All time series and map data are available and will be deposited in a dedicated archive (data publication). Existing web links are provided for all publicly available datasets (CCIEA, NOAA ERDDAP and Axiom). There are no restrictions on data availability and are available from the corresponding author.

Field-specific reporting

Please select the one below that is the best fit for your research. If you are not sure, read the appropriate sections before making your selection.

☐ Life sciences ☐ Behavioural & social sciences ☒ Ecological, evolutionary & environmental sciences

For a reference copy of the document with all sections, see nature.com/documents/nr-reporting-summary-flat.pdf

Ecological, evolutionary & environmental sciences study design

All studies must disclose on these points even when the disclosure is negative.

Study description	Observational data were collected during an annual fisheries survey. Additional data on oceanographic conditions were derived from a data-assimilative (integrates satellite ocean conditions) oceanographic model that is publicly available. Data on fishery landings and numbers of whale entanglements were derived from publicly available state and federal government databases. Time series catch data for forage species are presented as standardized anomalies. Number of annual confirmed whale entanglements and fishery landings data are presented in their original units. Acoustic data on krill distribution and abundance are examined for clustering using a Moran's I test for describing their spatial intensity.
Research sample	There are 3 sample units: (1) mid-water trawl sampling station event; used to summarize the 'catch-per-unit-effort' of species abundance and species richness (number of taxa present) per trawl, (2) acoustic index of the relative abundance of euphausiids (krill) per nautical mile (nautical area scattering coefficient), and (3) sighting number of whales per 3 km interval.
Sampling strategy	Approximately 120 mid-water trawls are collected per survey and depending on weather, typically each sampling station or area is occupied 2-3 times per survey (standardized since 1983) both on and off the continental shelf. Acoustic data and visual surveys for seabird and whales are collected continuously during daylight hours. All samples were used in the analysis in order to describe the ecosystem state. Due to the size and previous assessment of the geospatial aspects of the acoustic data set, additional data aggregation involved the summarization of acoustic values per 25 km2.
Data collection	Fisheries data were collected aboard a research vessel. All mid-water trawling was conducted at night following standardized protocol. Long-term (since 1983) sampling stations are fixed and located throughout the California Current Ecosystem. A team of fisheries scientists collected trawl data and identified all taxa immediately after each trawl. Data were entered into a relational database to assure data quality. Sightings data on marine mammals were collected by a trained observer and entered in real time using a sightings database.
Timing and spatial scale	The fisheries survey occurs each year from May-June and examines the mesoscale (1000s km) dynamics of biological and physical oceanography for the California Current Large Marine Ecosystem.
Data exclusions	No data were excluded from the analysis.
Reproducibility	All fisheries catch data are derived from observations and details are provided on how they were organized. The NOAA California Current Integrated Ecosystem Assessment stores all observation data and describes their reproducibility.
Randomization	Not relevant to the study given the nature of summarizing fisheries survey and oceanographic data.
Blinding	Blinding is not relevant to the study. All observation data were collected and organized following standardized protocol.

Did the study involve field work? ☒ Yes ☐ No

Field work, collection and transport

Field conditions	Research was conducted during the annual NOAA-NMFS Rockfish Recruitment and Ecosystem Assessment Survey involving the use of a U.S. Government Fisheries Science Vessel. The vessel is equipped with trawling gear, acoustics and a variety of sensors to monitor oceanographic conditions (e.g., temperature, salinity, bathymetry, acoustic signals). Sightings surveys of whales were collected using standardized visual techniques.
Location	California Current Large Marine Ecosystem; latitudes 40N-32N; mid-water trawl surveys were conducted at 30-40 m depth and occurred in water depths ranging from 50-4000 m, that span the continental shelf to beyond the outer slope.
Access and import/export	All data collection at sea was approved by NOAA and all permits were obtained.
Disturbance	Standardized marine mammal watches were conducted prior to all mid-water trawling stations and trawling was halted if any marine mammals occurred within 1 nautical mile of sampling locations.

Reporting for specific materials, systems and methods

We require information from authors about some types of materials, experimental systems and methods used in many studies. Here, indicate whether each material, system or method listed is relevant to your study. If you are not sure if a list item applies to your research, read the appropriate section before selecting a response.

Materials & experimental systems

n/a	Involved in the study
☒	☐ Antibodies
☒	☐ Eukaryotic cell lines
☒	☐ Palaeontology
☐	☒ Animals and other organisms
☒	☐ Human research participants
☒	☐ Clinical data

Methods

n/a	Involved in the study
☒	☐ ChIP-seq
☒	☐ Flow cytometry
☒	☐ MRI-based neuroimaging

Animals and other organisms

Policy information about studies involving animals; ARRIVE guidelines recommended for reporting animal research

Laboratory animals	N/A
Wild animals	Mid-water trawl catch data involves the take of small fish and invertebrates species (micronekton <400mm). These include, euphausiids, young of the year rockfish (Sebastes spp.), anchovy, sardine and squids. Organisms generally do not survive sampling using mid-water trawls; if they do survive they are released back to the ocean, but additional samples are preserved (frozen) for a variety of institutional studies.
Field-collected samples	No laboratory work was conducted nor were the use of transported organisms off site.
Ethics oversight	The fisheries research was approved by the U.S. Government and met all ethical oversight.

Note that full information on the approval of the study protocol must also be provided in the manuscript.
